# Assessment of data processing to improve reliability of microarray experiments using genomic DNA reference

**DOI:** 10.1186/1471-2164-9-S2-S5

**Published:** 2008-09-16

**Authors:** Yunfeng Yang, Mengxia Zhu, Liyou Wu, Jizhong Zhou

**Affiliations:** 1 Biosciences Division, Oak Ridge National Laboratory, Oak Ridge, TN 37831, USA; 2 Computer Science Department, Southern Illinois University, Carbondale, IL, USA, 62901, USA; 3 Institute for Environmental Genomics, and Department of Botany and Microbiology, University of Oklahoma, Norman, OK 73019, USA

## Abstract

**Background:**

Using genomic DNA as common reference in microarray experiments has recently been tested by different laboratories. Conflicting results have been reported with regard to the reliability of microarray results using this method. To explain it, we hypothesize that data processing is a critical element that impacts the data quality.

**Results:**

Microarray experiments were performed in a γ-proteobacterium *Shewanella oneidensis*. Pair-wise comparison of three experimental conditions was obtained either with two labeled cDNA samples co-hybridized to the same array, or by employing *Shewanella *genomic DNA as a standard reference. Various data processing techniques were exploited to reduce the amount of inconsistency between both methods and the results were assessed. We discovered that data quality was significantly improved by imposing the constraint of minimal number of replicates, logarithmic transformation and random error analyses.

**Conclusion:**

These findings demonstrate that data processing significantly influences data quality, which provides an explanation for the conflicting evaluation in the literature. This work could serve as a guideline for microarray data analysis using genomic DNA as a standard reference.

## Background

DNA microarray technology has been quickly adapted by mainstream laboratories to explore gene expression profiles of part or whole-genome for an organism [1,2]. A number of microarray studies use an experimental design in which experimental and reference RNA samples are transcribed into cDNA molecules, labeled with different fluorescent dyes (typically Cy5 and Cy3) and co-hybridized to the same microarray slide [3]. This approach, sometimes called type 1 approach [4], is very costly and tedious for a large number of samples, for which comparison across all samples are often desired. Mathematic calculation reveals that pairing all of the possible pairs for *n *samples results in a total of n*(*n*-1)/2 combinations. This polynomially increasing number could become unmanageable for individual laboratory when *n *is a big number. In addition, it is nearly impossible to compare data across experiments since the cDNA reference sample composition is subjected to differences of experimental design and hence not universal. It has been desired for a long time to develop novel strategies to integrate data across multiple, initially unrelated studies between laboratories or over a long period of time to promote data sharing and integration. Lastly, this approach provides no information on the absolute mRNA abundance, which is often useful since it has been established in that global transcriptional levels in microorganisms have strong correlation with global protein levels and gene essentiality [4-6].

A conceptually sound solution to the problems in type 1 experiments is to use "reference design", which requires co-hybridization of a common reference with all of the samples of the microarrays. Typically, the ratio (γ1) from cDNA: common reference is compared to another ratio (γ2) from cDNA: common reference. The computed "ratio of ratios" (γ1/γ2) is considered to be equivalent to direct cDNA: cDNA comparisons. In contrast to the type 1 approach, this "reference design" approach is called type 2 approach [4], in which only *n *microarrays are needed to calculate the ratios of any possible pairs of *n *samples. Apparently, this strategy greatly reduces the costs and time incurred in type 1 experiments. In addition, the absolute mRNA abundance of each gene could be deduced from γ1 and γ2, when the copy number of each gene is known for the common reference.

An ideal reference should fulfill the criteria of universality, reproducibility and uniformity, meaning that it should be universal across diverse microarrays, reproducible over a long time frame and in different laboratories, and represents each gene at a uniform level. One kind of such references is common RNA pools assembled from a number of different cell lines, tissues and conditions. Commercial universal RNA references are now available for mouse and human samples (Stratagene). However, the RNA references fall well short of the aforementioned criteria. Although RNA pools are more comprehensive than a single source of RNA sample, it still partially represents the whole genome; there is inherent biological variability among different RNA samples; and RNA could be degraded over time. Therefore, data quality across multiple studies is inevitably compromised. To address these issues, genomic DNA has been proposed to replace universal RNA reference [7]. It is easy and economic to prepare genomic DNA in large amount with low variations between different laboratories. Furthermore, genomic DNA is stable and could be stored over a long period of time. It is independent of variations from one preparation to another, which is a desirable feature of universal reference. In addition, genomic DNA represents entire genome completely and uniformly, since the majority of genes are presented in single or double copies in the genomes. It is especially useful for microbial functional genomics because of low representation of repetitive sequences and intergenic regions in the genome. In addition, this feature makes it easy to profile absolute mRNA levels. Several recent studies have proven that genomic DNA reference is indeed very effective and faithful for gene expression profiling [8-14]. Furthermore, a comparative study between genomic DNA reference and universal RNA reference has reached the conclusion that genomic DNA is superior for routine use [11].

Nevertheless, adopting genomic DNA as reference also creates new challenges. It is conceivable that though this strategy enables the integration of disparate studies, it brings in new variations. For example, spots with low signal intensity from labeled genomic DNA are prone to high standard errors for measurements, and spots with high intensity considerably interfere with the hybridization of cDNA samples to the probes, leading to low fidelity in the ratio of cDNA to genomic DNA. For quality control purpose, it is critical to identify these variances and remove ambiguous values by data analyses. However, to our best knowledge, so far this problem has not been unequivocally tackled and there is no consensus among the scientific community for the data analyses of microarray using genomic DNA reference. For instance, some researchers conducted array-to-array comparison with little data processing except for background subtraction and removal of poor or negative spots [15,16], while the others employed extensive techniques involving complicated statistical models [8,9,13,14]. It is thus necessary to appraise the performance of different data processing techniques.

In this study, we address this need by conducting a comparative study of type 1 and 2 experiments in a γ-proteobacterium *Shewanella oneidensis*, which was capable of respiring with oxygen, fumarate, trimethylamine-N-oxide (TMAO), manganese (IV) oxides and ferric oxides as terminal electron acceptors [17-19]. Gene expression profiles of *S. oneidensis *were generated under three growth conditions – aerobic growth or anaerobic growth with fumarate or ferric citrate as electron acceptor. Variations among gene expression profiles were compared and we concluded that data processing techniques, including setting minimal number of replicates, logarithmic (log) transformation and random error analyses, appeared to be valuable to improve data quality.

## Results

As indicated in the introduction, type 2 experiments using genomic DNA reference could add an additional layer of variance and hence impair data quality as compared to type 1 experiments. To evaluate it, we performed both type 1 and 2 microarray experiments. RNA was extracted from mid-logarithmically grown *Shewanella oneidensis *strain DSP10 under aerobic condition (O_2_), or under anaerobic conditions with fumarate (Fum) or ferric citrate (Fe) as electron acceptors. cDNA was subsequently transcribed and labeled with Cy5 or Cy3, and any pair of two conditions was co-hybridized on microarray slides, yielding three direct ratios, namely Fum/O_2_, Fe/O_2 _and Fe/Fum. Meanwhile, RNA from each condition was reversely transcribed and labeled by Cy5 and co-hybridized with Cy3-labeled *Shewanella *genomic DNA. To obtain expression ratios of Fum/O_2_, Fe/O_2 _and Fe/Fum, the ratios of cDNA: gDNA were calculated, and then the inferred (indirect) ratios were obtained by calculating the "ratio of ratios" as (cDNA1/gDNA) over (cDNA2/gDNA). Results from type 1 experiments were compared to those obtained in type 2 experiments. Two previous studies employed few data processing techniques except for basal ones such as background subtraction and removal of poor or negative spots [15,16]. Therefore, the same procedures were applied to generate the inferred ratios. Two criteria were used to judge the similarity between both methods. First of all, the overall similarity was determined by correlation coefficient derived from both sets of expression ratios over the entire genome, which provides a comprehensive view of the impact when a data processing technique is evaluated. Secondly, to identify the most inconsistent data, the changes of gene expression were categorized as "induction (ratio > 2)", "repression (ratio < 0.5)" and "no change (0.5 <= ratio <= 2). For example, if the result is 3 for type 1 experiments but 30 for type 2 experiments, the data can still be considered as consistent from biological point of view since the gene is induced in both experiments. However, if the result is 3 for type 1 experiments but 0.3 for type 2 experiments, they should be considered as inconsistent because they represent two opposite categories as induction and repression, respectively. In this study, we focus on this type of inconsistency because they have the greatest impact on the biological interpretation.

**Figure 1:**
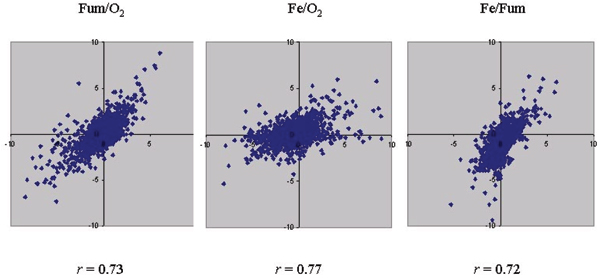
Scatter plot represents the correlation of the results from type 1 and 2 experiments. Genes with expression changes in opposite categories (induction vs. repression) in both approaches are visualized as dots located in the 2nd and 4th quadrants and away from the origin. Pearson's correlation coefficients (*r*) are indicated in each panel.

The Pearson correlation coefficients of these two results fell in the range of 0.72–0.77 (Figure 1). In contrast, the average correlation coefficient of replicates within type 1 or 2 experiments was 0.87. Therefore, the results from type 1 and 2 experiments were not very similar. In addition, a number of ratios from two methods (11 values for Fum/O_2_, 17 values for Fe/O_2 _and 8 values for Fe/Fum) fell into two opposite categories (induction vs. repression) (Figure. 1). Therefore, there were clear inconsistencies between type 1 and 2 experiments.

To provide quantitative evaluation on the consistency from statistical viewpoint, one-way ANalysis Of VAriance (ANOVA), a powerful statistical approach to determine differences, was applied to selected genes. Table 1 shows two representative genes with multiple replicates for the expression ratios of Fum/O_2_. Analysis of OmcA leads to the *p *value of 0.0198, inferring that two reference methods are significantly different as judged by significance level of 0.05. In contrast, the *p *value is 0.5054 for gene NapG, which fails to reject the null hypothesis.

**Table 1:** ANOVA tests for napG and omcA.

NapG	N	Means	SD	SE	
CDNA	12	-3.234	1.627	0.4698	
GDNA	10	-2.674	2.241	0.7087	
Source Variance	SSq	DF	MSq	F	*p*
NapG	1.709	1	1.709	0.46	**0.5054**
Within-cells	74.338	20	3.717		
					
OmcA	N	Means	SD	SE	

CDNA	12	1.667	0.527	0.1521	
GDNA	2	0.461	1.047	0.7400	
Source Variance	SSq	DF	MSq	F	*p*
omcA	2.494	1	2.494	7.21	**0.0198**
Within-cells	4.149	12	0.346		

N: number of replicates; SD: standard deviation; SE: standard error; SSq: sum of square; DF: degree of freedom; MSq: mean square; F: F test value; and *p*: probability value.

A previous study has identified a number of genes previously regulated under Fum and Fe-reducing conditions in *S. oneidensis *[20]. While the results of type 1 experiments were generally consistent with existing knowledge, the results of type 2 experiments were not. For example, it is known that *c*-type cytochromes OmcA and OmcB exist as a complex on outer membrane and function to reduce extracellular Fe(III) and U(VI) as terminal electron acceptors. Their expression is induced for several folds under anaerobic conditions ([21] and unpublished results in our laboratory). In addition, expression of fumarate reductase FccA and its paralog IfcA is induced under Fe-reducing condition [21,22]. All of these expression patterns have been correctly confirmed by type 1 but not type 2 experiments (Table 2). Therefore, the basal data analyses are not efficient to remove potentially noisy values in type 2 experiments.

**Table 2:** The results of type 1 and 2 microarray experiments for genes encoding cytochrome *c*.

	**Fum/O**_2_		**Fe/Fum**		**Fe/O**_2_	
Gene	Type 1	Type 2	Type 1	Type 2	Type 1	Type 2

ifcA-1	N/A	**1.79**	**4.70**	**2.06**	**4.36**	**1.15**
ifcA-2	**4.16**	**8.52**	**24.16**	**19.88**	**7.87**	**2.33**
SO1427	**2.93**	**5.4**	**18.35**	**12.81**	4.19	2.37
MtrB	**2.81**	**6.15**	3.56	1.98	1.56	0.32
MtrA	**3.83**	**2.53**	2.51	2.64	1.36	1.04
OmcB	N/A	**0.75**	1.40	0.89	1.30	1.19
OmcA	**2.61**	**0.96**	1.70	1.11	1.41	1.15
MtrF	1.24	2.06	1.97	1.15	1.66	0.56
SO1781	0.92	1.84	1.15	0.48	1.16	0.26
MtrD	N/A	1.96	N/A	0.30	N/A	0.15
FccA	1.50	1.25	**2.34**	**0.79**	1.68	0.63
NapB	**0.26**	**0.36**	0.66	0.61	3.85	1.68
NapH	**0.1**	**0.28**	1.07	0.21	3.23	0.77
NapG	**0.17**	**0.24**	0.54	0.38	5.28	1.61

Values in boldface are consistent with previous reports, while values underlined are not. N/A: data not available.

### The minimal number of replicates

**Figure 2:**
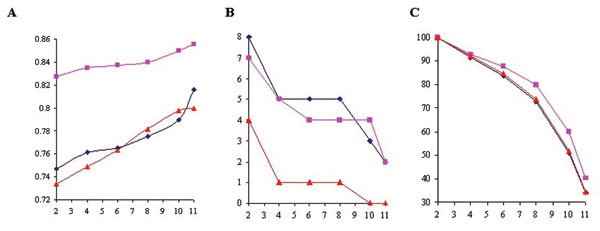
Assessment of minimal number of replicates. Blue line: Fum/O_2_; pink line: Fe/O_2_; and red line: Fe/Fum. X-axis: minimal number of replicates. (A) Plot of *r *values with different minimal number of replicates. Y-axis: *r *values comparing the results from type 1 and 2 approaches. (B) Number of genes in opposite categories (induction vs. repression) with different minimal numbers of replicates. Y-axis: numbers of genes. (C) Total number of genes with different minimal numbers of replicates. Total number of genes was set to 100% when minimal number of replicates was 2, and the total number of genes at other minimal number of replicates was normalized accordingly. Y-axis: numbers of genes.

Minimal number of replicates serves as a threshold to remove genes without sufficient number of observations. If the number is lower than the threshold, the data for that gene are then disregarded. We first tested whether setting a minimal number of replicates improves the quality of the data. Pearson correlation coefficient (*r*) of both approaches were increased under all pairs of conditions when the minimal number of slides was set higher, demonstrating that it significantly improves data quality at global level (Figure 2A). Furthermore, the inconsistency between both approaches, as represented by the number of genes in opposite categories, was also reduced (Figure 2B). However, this is at the expense of losing significant amount of data (Figure. 2C). In this study, over 60% of values were lost when the minimal number of replicates was set to be 11.

### Logarithmic transformation

**Figure 3:**
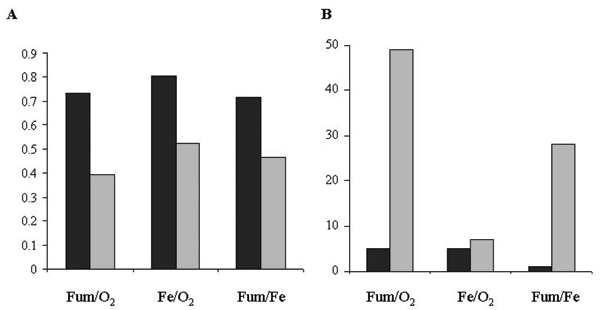
Assessment of logarithmic transformation. Black and gray columns represent proportional and additive models, respectively. (A) A histogram representing *r *values. (B) A histogram showing the number of genes in opposite categories.

If there is a positive relationship between the standard deviation (SD) of the replicates and their mean, logarithmic transformation is often conducted to remove a large portion of the relationship between the SD and mean. This approach is called proportional model. If there is no positive relationship between SD and mean, no log transformation should be applied and the data are analyzed in the raw form. This is called additive model. It is interesting to test whether applying logarithmic transformation has an impact in microarray analyses. Figure 3A indicated that proportional model was clearly superior to additive model in our data sets. Applying proportional model resulted in *r *values of 0.73, 0.80 and 0.72 for Fum/O_2_, Fe/O_2 _and Fe/Fum conditions, respectively. In contrast, applying additive model resulted in much lower *r *values in the range of 0.39–0.53. Furthermore, proportional model resulted in fewer genes in opposite categories than additive model (Figure 3B). To explain it, correlation coefficient of SD and mean value was calculated for each microarray dataset. There was clear positive relationship between SD and mean, as indicated by correlations of 0.81–0.88. These results suggested that logarithmic transformation should be applied.

### Random error analyses

No measurement is entirely accurate. It is hence important to estimate the amount of measured value that could randomly deviate from the true value. This technique is called random error analyses or uncertainty analyses. A series of repeated measurements are usually used to make a reasonable estimate. The repeated measurements include biological and technical replicates. It is necessary to take random error into account to determine the significance of the results. One method, called small sample method, estimates random error on the replicates of individual genes, regardless of all of the other genes in the array. In contrast, random error could also be estimated on the entire array because this might be more accurate than estimation of small number of replicates for individual genes. A common error approach makes the assumption that the SD of replicates is unrelated to mean signal intensity. Alternatively, a curve fit approach could recognize the relationship between SD and mean by a regression line (curve fit).

**Figure 4:**
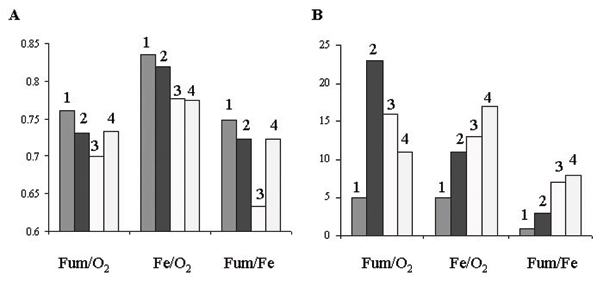
Assessment of random error analyses. Column 1: small sample method; 2: Curve fit method; 3: common error method; and 4: no random error analyses. (A) A histogram representing *r *values. (B) A histogram showing the number of genes in opposite categories.

Figure 4 demonstrates that small sample method has the best outcome, as judged by the highest *r *values (Figure 4A) and the fewest genes in opposite categories (Figure 4B). Moreover, it is better than cases without random error analyses, suggesting that applying small sample method improves data quality. In contrast, common error method yields the lowest *r *values and the most genes in opposite categories. This observation is consistent with our discovery of positive relationship between SD and mean in the dataset.

### Other data processing techniques

Outliers are data points that are not faithfully reproducible among replicates, yet have a disproportionately large effect on the average values. Thus removal of outlier from source file is likely to improve data quality. Hence we compared results with and without outlier removal. Two criteria, namely *p *< 0.05 and *p *< 0.01, were applied to outlier removal. *p *refers to the possibility of making a Type I error in identifying outliers. Accordingly, *p *< 0.05 will detect more outliers than *p *< 0.01. Table 3 shows that the consistency between type 1 and 2 approaches is not improved by removing outliers, as demonstrated by little changes of *r *values and number of genes in opposite categories. Notably, when outlier removal was tested, minimal number of replicates was set as 4. It is thus possible that signal fluctuation is already fairly limited. Indeed, at outlier filter of *p *< 0.05, only ~15 values (i.e. 0.3% of total values) were removed from each of Fum/O_2_, Fe/O_2 _and Fe/Fum comparisons. If minimal number of replicates is not used, outlier removal has a slight impact on data quality (data not shown).

**Table 3:** Assessment of outlier removal and flooring.

**Techniques**	***r***			**Number of genes in opposite categories**		
	Fum/O_2_	Fe/O_2_	Fe/Fum	Fum/O_2_	Fe/O_2_	Fe/Fum

No_outlier	0.76	0.84	0.76	5	1	1
Outlier_*p *< 0.01	0.76	0.84	0.75	4	3	1
Outlier_*p *< 0.05	0.76	0.84	0.75	5	5	1
No floor	0.76	0.85	0.77	2	6	2
Floor of 20	0.76	0.84	0.75	5	5	1
Floor of 50	0.77	0.84	0.78	5	4	1
Floor of 1%	0.75	0.83	0.72	7	7	2

Both techniques are dispensable for type 2 microarray analyses.

We also tested the effect of flooring. Low signals in direct comparison of RNA samples often produce spurious expression ratios, thus signals below a certain threshold level are often set to the threshold level [23,24]. To test if it is necessary to do so, different flooring strategies were employed: no floor, floor an absolute value of 20 or 50, or floor 1% lowest signals. As shown in Table 3, data quality is not improved, indicating that flooring does not appear to be an effective technique in type 2 experiments.

## Discussion

It is often desirable to compare results from any two experimental conditions in microarray studies. Type 2 microarray experiments using a common reference such as genomic DNA allows for inter-conditional comparisons. However, the reliability of the comparison is often questionable since microarray is notorious for its considerable fluctuation of signals. In this report, we test a variety of data processing techniques in order to improve data quality in type 2 experiments. Two criteria are used to evaluate these techniques: by (1) correlation coefficient to the corresponding type 1 experiments; and (2) classifying differential expression values of genes into "up", "down" and "constant" categories, and then focus on genes in opposite categories. The first criterion evaluates the impact of techniques at the whole-genome level, while the latter addresses the most inconsistent data. Two-fold was used as threshold to classify the categories, which was reported to be a solid benchmark for induction or repression of gene expression [25]. However, it is still likely that ratio changes of less than two fold are both statistically significant (judged by *z*-test or *t*-test) and biologically meaningful. Thus two-fold is used here as a general guideline to simplify our study.

One important prerequisite in our study is that the results from type 1 experiments are more reliable than those from type 2 experiments. One way to comprehend it is to analogize with triangle inequality relation for metric spaces: errors of an indirect path should be no less than errors of a direct path. To date, the reliability of data in type 1 experiments has been extensively studied. It has been estimated that over 90% of the results could be verified by other techniques such as quantitative reverse transcription PCR or northern blot [26]. Therefore, it is reasonable to believe that the results of type 2 experiments will be more reliable when their consistency to those of type 1 experiments is improved. This is also confirmed by the existing information of gene expression ratios, as exemplified in Table 2.

Among data processing techniques, the minimal number of replicates seems to be a critical one to filter out inconsistency between the results of type 1 and 2 experiments, as demonstrated in Figure 2. Consistently, previous studies adopting this technique concluded that the results of type 2 experiments were fairly reliable [8,9], while other studies without using it gave unfavorable opinion to the reliability of microarray data using genomic DNA reference [15,16].

A large number of data processing strategies are available for microarray data analyses. In this short report, we could only examine several of them. Moreover, caution should be taken to extend conclusions from our study to other microarray experiments. It is likely that some of our conclusions would not hold for specific microarray datasets. Nevertheless, since we show here that data analysis process has a significant impact on the reliability of the results in type 2 experiments, it is thus advisable for researchers to evaluate their data processing techniques carefully when genomic DNA or another common reference is used in microarray experiments.

## Materials and methods

### Sample preparation and microarray scanning

*Shewanella oneidensis *whole-genome microarray was constructed as described previously [27]. Strain DSP10, a rifampin-resistant derivative of strain MR-1, was used in this study because this strain has been widely used in genetic studies of *Shewanella oneidensis*. It is thus of interest to catalog DSP10's gene expression.

DSP10 was grown aerobically in 100 ml Luria-Bertani medium (LB, Difco) to mid-logarithmic phase at 30°C. Alternatively, DSP10 was grown anaerobically to mid-logarithmic phase in 200 ml LB liquid supplemented with 20 mM lactate, and with either 10 mM fumarate or 10 mM ferric citrate as electron acceptor. Mid-logarithmic phase was determined by measuring the turbidity at 600 nm in a spectrophotometer for aerobic or anaerobic 10 mM fumarate cultures, or by epifluorescence microscopy using acridine orange staining [28] for anaerobic 10 mM Fe(III) citrate cultures. Cells were then collected by centrifugation at 4 krpm for 10 minutes. After discarding the supernatant, the pellets were immediately lysed by Trizol (Invitrogen), or chilled in liquid nitrogen and then kept at -80°C for later use. Total RNA was extracted as described previously [29]. RNA samples were treated with RNase-free DNase I (Ambion) to digest residual chromosomal DNA and then purified with RNeasy Kit (Qiagen) prior to spectrophotometric quantification at 260 and 280 nm. For type 1 experiments, cDNA was produced in a first-strand reverse transcription (RT) reaction and labeled with Cy5 or Cy3 dUTP (Amersham Biosciences) in the presence of random hexamer primers (Invitrogen). Fluorescein labeled probes were then purified using a PCR purification kit (Qiagen). Slides were pre-hybridized at 50°C for about one hour to remove unbound DNA probes in a solution containing 50% (V/V) formamide, 9% H_2_O, 3.33% SSC (Ambion), 0.33% sodium dodecyl sulfate (Ambion), and 0.8 μg/μL bovine serum albuminin (New England Biolabs). Slides were hybridized at 50°C over night with Cy5- and Cy3-labeled probes in the above solution, minus BSA and with the addition of 0.8 μg/μL herring sperm DNA (Invitrogen) to prevent random binding. Pre-hybridization and hybridization were carried out in hybridization chambers (Corning). Slides were then washed on a shaker at room temperature in the following order: 7 minute in 1 × SSC, 0.2% SDS; 7 minute in 0.1 × SSC, 0.2% SDS; and 40 second in 0.1 × SSC. For type 2 experiments, 100 ng *S. oneidensis *genomic DNA (gDNA) was amplified by incubated at 37°C for 3 hours using Klenow fragment of DNA polymerase (Invitrogen) and random primers followed by transferring on ice to stop the labeling. Cy3 dUTP was incorporated in the product (Amersham Biosciences) and then Cy3-labeled genomic DNA was co-hybridized with Cy5-labeled cDNA on pre-hybridized microarray slides as described above. A total of twelve replicates were prepared for both approaches. A program ImaGene version 5.5 (Biodiscovery) was used to grid and quantify microarray images. Background signals around each spot were calculated and subtracted from the signal intensity of each spots. Spots of Signal/background ratios < 3 were regarded as negative spots. All negative, poor and empty spots were flagged and discarded.

### Data analysis of type 1 experiments

Data analysis of type 1 experiments in *S. oneidensis *was carried out as previously described [29]. Quantified microarray was loaded onto GeneSight-Lite, a plug-in program of ImaGene 5.5 for background subtraction, flagged spots removal, floor of 20 and normalization by mean. The results after processing were subsequently transferred onto software ArrayStatTM (Imaging Research), in which extensive statistical tools were available. In general, minimal number of replicates was set as 4, proportional model and small sample model were selected before outlier removal at *p *< 0.05. The significance of differential expression was determined by two-way *t*-test.

### Data analysis of type 2 experiments

Local background subtraction and flagged spots removal were implemented in the same way as type 1 experiments. If no other data processing technique was used, inferred ratio was calculated by T_2_/T_1 _= (T_2_/R_2_)/(T_1_/R_1_), where T and R represented the mean value of cDNA and genomic DNA reference signals from all of the twelve replicates, respectively. To evaluate selected data processing techniques, data were processed in the same order as type 1 experiments, with change in only one parameter for each time. Certain parameters were specified as: floor of 20 and normalization by mean, data with less than the minimal number of replicates of 4 were removed, and then followed by execution of proportion model and small sample model. Then outlier was removed by *p *< 0.05 and finally, expression ratios were obtained by calculating the division of two ratios.

### Pearson correlation coefficient, the number of genes in opposite categories and one-way ANOVA

Pearson correlation coefficient (*r*) was computed between results acquired from type 1 and 2 approaches. To obtain the number of genes in opposite categories, two-fold change was used as criterion. We consequently categorized the differential expression values into three classes: "up" for expression ratios of more than 2, "down" for expression ratios of less than 0.5, and "no change" for all other ratios. A gene was considered to be in opposite categories if its expression ratio was classified as "up" in type 1 experiment and "down" in type 2 experiment, or vice versus. For one-way ANOVA, the logarithmic transformation was applied to the ratio values to normalize the expression variation among genes and equalize the data scale intervals for ANOVA test. The significance level of *p *value < 0.05 was used as criterion to reject or accept the null hypothesis "the two reference methods are not significantly different from each other".

## Competing interests

The authors declare that they have no competing interests.

## Authors' contributions

YY contributed to the experimental design, conduction and analysis of the microarray experiments and manuscript writing. MZ and LW contributed to the evaluation of microarray data processing techniques. JZ contributed to the experimental design and manuscript revision. All authors read and approved the final manuscript.
